# Low physical activity and risk of falls among older adults in a high-altitude district of Peru: a secondary analysis of the Aunqui-Andes study

**DOI:** 10.1186/s12877-026-07909-6

**Published:** 2026-07-09

**Authors:** Jesus A. Huaracayo-Campos, Camila Rojas-Astorayme, Vilma Y. Rojas-Carranza, Leslie Salazar-Talla, Ana L. Alcantara-Diaz, Guido Bendezu-Quispe, Diego Urrunaga-Pastor, Fernando M. Runzer-Colmenares, José F. Parodi

**Affiliations:** 1 Carrera de Medicina Humana, Facultad de Ciencias de la Salud, Universidad Científica del Sur, Lima, Peru; 2 Escuela de Medicina Humana, Universidad Señor de Sipán, Chiclayo, Peru; 3 CHANGE Research Working Group, Carrera de Medicina Humana, Escuela de Medicina, Universidad Científica del Sur, Carretera Panamericana Sur 19, Distrito de Villa El Salvador, Lima, 15067 Peru; 4 Facultad de Medicina Humana, Centro de Investigación del Envejecimiento (CIEN), Universidad de San Martin de Porres, Lima, Peru

**Keywords:** Motor Activity, Accidental Falls, Aged, Rural Population, Altitude, Peru

## Abstract

**Background:**

Falls are the second leading cause of unintentional injury deaths and disproportionately affect older adults, especially in low- and middle-income countries. In Peru’s high-Andean rural settings, hypobaric hypoxia, rugged terrain, and limited healthcare may increase the risk of falls. Low physical activity is associated with the risk of falls, yet evidence from high-altitude rural populations is scarce. This study aimed to estimate the association between low physical activity and risk of falls among older adults in a high-Andean district of Peru.

**Methods:**

We conducted a secondary analysis of an analytical cross-sectional study (Aunqui-Andes) including 271 adults aged ≥ 60 years from Totos District, Ayacucho, Peru (3,286 m above sea level), enrolled in August 2022. Individuals with severe cognitive impairment or dependence for ambulation were excluded. Low physical activity was assessed using sex-specific cutoffs of the Physical Activity Scale for the Elderly (PASE), and risk of falls was defined as a Functional Reach Test distance < 20.32 cm. We fitted Poisson generalized linear models with a log link and robust variance to estimate adjusted prevalence ratios (aPRs) and 95% confidence intervals (95% CI), adjusting for sex, age group, marital status, educational level, comorbidities, and cognitive impairment.

**Results:**

We analyzed 271 older adults (median age 75 years; 59.8% women). The proportion classified as at risk of falls was 57.2% (*n* = 155), and the prevalence of low physical activity was 6.6% (*n* = 18). In crude models, low physical activity was associated with a higher risk of falls (cPR = 1.62; 95% CI: 1.33–1.97; *p* < 0.001). In multivariable models adjusting for sex, age group, marital status, educational level, comorbidities, and cognitive impairment, the association persisted (aPR = 1.32; 95% CI: 1.09–1.62; *p* = 0.007).

**Conclusions:**

Low physical activity was associated with a higher risk of falls among older adults living in a high-Andean district of Peru. These findings support incorporating practical screening for risk of falls using PASE and the Functional Reach Test, along with simple, locally adapted physical-activity support, into primary care and community programs in rural high-altitude areas and may inform public health actions to reduce fall-related harms in these underserved communities.

## Introduction

Falls represent the second leading cause of unintentional injury deaths worldwide. The World Health Organization estimates that approximately 684,000 individuals die annually from falls, with over 80% of these fatalities occurring in low- and middle-income countries. Adults aged 60 years and older experience the highest burden of fatal falls. Each year, 37.3 million falls are severe enough to require medical attention [1, 2]. Beyond mortality, falls result in fractures, social isolation, fear of falling (FoF), depression, and loss of independence in activities of daily living, posing a critical public health challenge [3, 4]. In Peru, this situation is particularly concerning as of the first quarter of 2020, 43.9% of households nationwide included at least one resident aged 60 years or older [5].

Low physical activity, defined as insufficient physical activity to meet current health recommendations [6], has been associated with a higher risk of falls, while higher activity levels are associated with lower levels risk [7]. In Peru, only 38.1% of the population meets international physical activity recommendations [8]. Low physical activity compromises quality of life by increasing susceptibility to obesity, type 2 diabetes mellitus (T2DM), high blood pressure (HBP), and Alzheimer’s disease, ultimately reducing life expectancy [6]. The global economic burden of low physical activity reaches US $47.6 billion annually in direct healthcare costs [9], while in Latin America and the Caribbean, it accounts for 0.53% of total healthcare expenditures [10]. Several validated instruments assess physical activity levels in older populations, including the International Physical Activity Questionnaire (IPAQ), the Physical Activity Scale for the Elderly (PASE), and accelerometers. These tools quantify activity time, metabolic task performance, and daily step counts [11–13].

Older adults residing in high-altitude rural areas present distinctive characteristics that may influence risk of falls and balance function. Chronic exposure to hypobaric hypoxia could generate neurological changes, particularly affecting executive functions and cognitive processing, as well as alterations in brain functional connectivity that may resemble accelerated aging processes. Additionally, altitude exposure may contribute to muscle mass loss through chronic tissue hypoxia, autonomic cardiovascular alterations, and in some cases, polycythemia that could affect cerebral and muscular oxygenation. These physiological adaptations, combined with the rugged terrain characteristic of Andean-rural communities and prevailing socioeconomic barriers, may configure a unique risk-of-falls profile among older adults in these regions [14, 15].

Systematic reviews and observational studies have demonstrated that low physical activity increases fall likelihood in older adults [16, 17]. However, these studies have not specifically examined high-Andean populations. In Peru, rural older adults constitute 13.3% of the elderly population, with at least one older adult present in 42.8% of rural households [18]. Access to health services remains severely limited in low-resource countries, particularly affecting older individuals living in poverty [19]. Educational attainment among rural older adults in Peru is notably low, especially among women [20]. Illiteracy rates reach 23.9% among older women overall, escalating to 54% in rural areas—threefold higher than among men [18]. Low educational levels perpetuate poor economic conditions, diminish health literacy, and restrict access to healthcare services regarding affordability, availability, and quality of care [21, 22]. Despite growing recognition of falls as a major health concern, the intersection of low physical activity and risk of falls in high-altitude older populations remains understudied. Investigating this association is essential to generate evidence that guides the development of targeted interventions aimed at improving living conditions and reducing risk of falls in these vulnerable populations. Therefore, this study aims to estimate the association between low physical activity and risk of falls among older adults residing in a high-Andean rural community, operationalized using the Functional Reach Test, a validated, objective measure of dynamic balance and an established proxy for fall risk in community-dwelling older adults [23, 24].

## Methods

### Study design, population, and sample

We conducted a secondary analysis of data from the Aunqui-Andes analytical cross-sectional study [25], which enrolled older adults in August 2022 in the district of Totos, at an altitude of 3,286 m above sea level (m.a.s.l.), Cangallo Province, Ayacucho Department, Peru. The cross-sectional design refers to the temporal framework of data collection, as all measurements were obtained at a single point in time. The primary study enrolled 292 adults aged 60 years or older without severe cognitive impairment or dependence for ambulation. For the present analysis, we excluded 21 participants with incomplete data on variables of interest, including the exposure, outcome, or prespecified covariates.

We implemented a district-wide census of adults aged 60 years or older residing in the following populated centers of Totos district: Totospampa, Chacabamba, Lloqllasqa, Quiñasi, Veracruz, Totos, Chuymay, Huanupampa, and Ramón Castilla, in coordination with the social development manager, municipal authorities, and community representatives. Recruitment was conducted through radio announcements, and the census achieved coverage of approximately 95% of the eligible older adult population in the district. All older adults who met the selection criteria and provided informed consent were enrolled. For the power calculation, we used proportions reported by Lewis et al. [26] 44.38% risk of falls among participants with low physical activity versus 28.37% among active participants, which yielded an estimated statistical power of 81.3% at a 95% confidence level in our sample of 271 older adults. This secondary analysis follows the Strengthening the Reporting of Observational Studies in Epidemiology (STROBE) recommendations for cross-sectional studies.

### Procedures

Data collection for the primary study involved planning visits to the populated centers of Totos district in coordination with the social development manager and community representatives of each municipality. The data collection team, composed of medical students and physicians under the supervision of the principal investigator, received standardized training in instrument application and data collection procedures. The assessments were carried out with the collaboration of Ayacuchan natives proficient in both Spanish and the Chanka variant of Quechua spoken in the Totos district. Furthermore, the research team possessed expertise in Quechua Chanka, ensuring culturally and linguistically appropriate data collection. Participant recruitment was conducted through radio announcements in the populated centers, and comprehensive medical evaluation campaigns were carried out in health facilities and populated centers targeting older adults.

Enrollment began when older adults who met the selection criteria voluntarily agreed to participate after the informed consent was read, explained, and accepted. Surveys and instruments were administered through individualized interviews. The assessments were carried out independently of the healthcare provided by the district health center, and this was communicated to the older adults during enrollment. No biological samples were collected in either the primary or the present study. All required instruments and materials for evaluating older adults were transported to the Totos district for the data collection campaigns.

### Variables

#### Outcome variable: risk of falls

Risk of falls was assessed using the Functional Reach Test. This test involved participants standing and reaching forward as far as possible without losing balance or moving their feet. We allowed two attempts, recording the maximum reach in centimeters. A measurement of less than 20.32 cm classified a participant as having an altered test, indicating risk of falls [23, 24].

#### Exposure variable: low physical activity

Low physical activity was assessed using the PASE. This validated questionnaire gathered detailed information on participants’ household activities, leisure time activities, and work-related activities over the preceding seven days. The PASE score was calculated by multiplying the time spent on each activity by its corresponding activity weight, then summing these products. Low physical activity was defined using sex-specific cutoffs: scores below 52 for women and below 64 for men, consistent with approaches used in similar geriatric populations [11, 12]. Low physical activity and sedentary behavior are related but distinct constructs. Low physical activity refers to insufficient engagement in moderate-to-vigorous activity to meet current health recommendations, whereas sedentary behavior refers to waking time spent in low-energy-expenditure activities such as sitting or watching television, regardless of overall activity level [27].

#### Other variables

##### Sociodemographic characteristics

We collected data on sex (female, male), age (analyzed as a continuous variable and categorized into two groups: 60–80 years and older than 80 years), marital status (single, cohabiting or married, widowed or divorced), and educational level (no education or incomplete elementary school, complete primary or incomplete secondary school, complete secondary school, technical or professional education).

##### Anthropometric measurements

We measured weight (kg) and height (cm) without shoes to calculate Body Mass Index (BMI, kg/m²). Body mass index was analyzed as a continuous variable (mean ± standard deviation).

##### Geriatric syndromes and functional assessments

We assessed medical comorbidities through self-report, including HBP, T2DM, chronic kidney disease (CKD), arthritis (including osteoarthritis and rheumatoid arthritis), hypercholesterolemia, gastritis, low back pain, and osteoporosis. Comorbidities were categorized as 0, 1, or ≥ 2 conditions [28].

Polypharmacy was defined as the consumption of three or more concurrently prescribed medications, excluding alternative medicine or nutritional supplements. Urinary incontinence was assessed using a specific question from the Edmonton Frail Scale: “Do you have problems holding your urine when you don’t feel like urinating?” with dichotomous response options (yes/no) [29, 30].

Cognitive impairment was evaluated using the Short Portable Mental Status Questionnaire (SPMSQ), a 10-item tool assessing long- and short-term memory, orientation, and details of daily activities. We adjusted SPMSQ scores according to participants’ educational levels: allowing one fewer error for those with higher education and one additional error for those with incomplete primary education or no formal education. Cognitive impairment was categorized as: no impairment (0–2 errors), mild impairment (3–4 errors), and moderate impairment (5–7 errors) [31, 32].

Depressive symptoms were assessed using the 5-item Geriatric Depression Scale (GDS-5). A score of two or more indicated the presence of depressive symptoms, consistent with validated thresholds for screening in geriatric populations [31, 33].

The risk of sarcopenia was assessed using the SARC-F instrument (Strength, Assistance in walking, Rise from a chair, Climb stairs, and Falls), where a score of four or more points indicated sarcopenia risk [34, 35].

### Statistical analysis

Data were tabulated in the Research Electronic Data Capture (REDCap) platform, downloaded in “.dta” format, and imported into Stata/SE version 16.0 (StataCorp, TX, USA) for statistical analysis. Descriptive statistics were calculated for qualitative variables as absolute and relative frequencies, and for quantitative variables as means with standard deviations or medians with interquartile ranges (25th and 75th percentiles) according to distributional assumptions.

Bivariate analyses compared proportions for categorical variables using Pearson’s chi-squared test or Fisher’s exact test based on assumption fulfillment. To compare quantitative variables by risk of falls, we used Student’s t-test or Mann-Whitney U test, depending on whether parametric assumptions were met.

To estimate the association between low physical activity and risk of falls, we constructed generalized linear models of the Poisson family with a log link function and robust variances, reporting crude prevalence ratios (cPR) and adjusted prevalence ratios (aPR) with their respective 95% confidence intervals (95% CI). The adjusted model included confounders identified a priori based on existing literature: sex, age group, marital status, educational level, comorbidities and cognitive impairment [23, 26, 36, 37]. In the multivariable model, the following reference categories were used: sex (male), age group (60–80 years), marital status (single), educational level (no education or incomplete elementary school), comorbidities (zero comorbidities), and cognitive impairment (no impairment). Multicollinearity was assessed using variance inflation factors (VIF), with values greater than 3 considered indicative of multicollinearity; no VIF values exceeded this threshold. Statistical significance was defined as a p-value less than 0.05.

## Results

### Descriptive and bivariate analysis according to the prevalence of low physical activity in the study sample

**Fig. 1:**
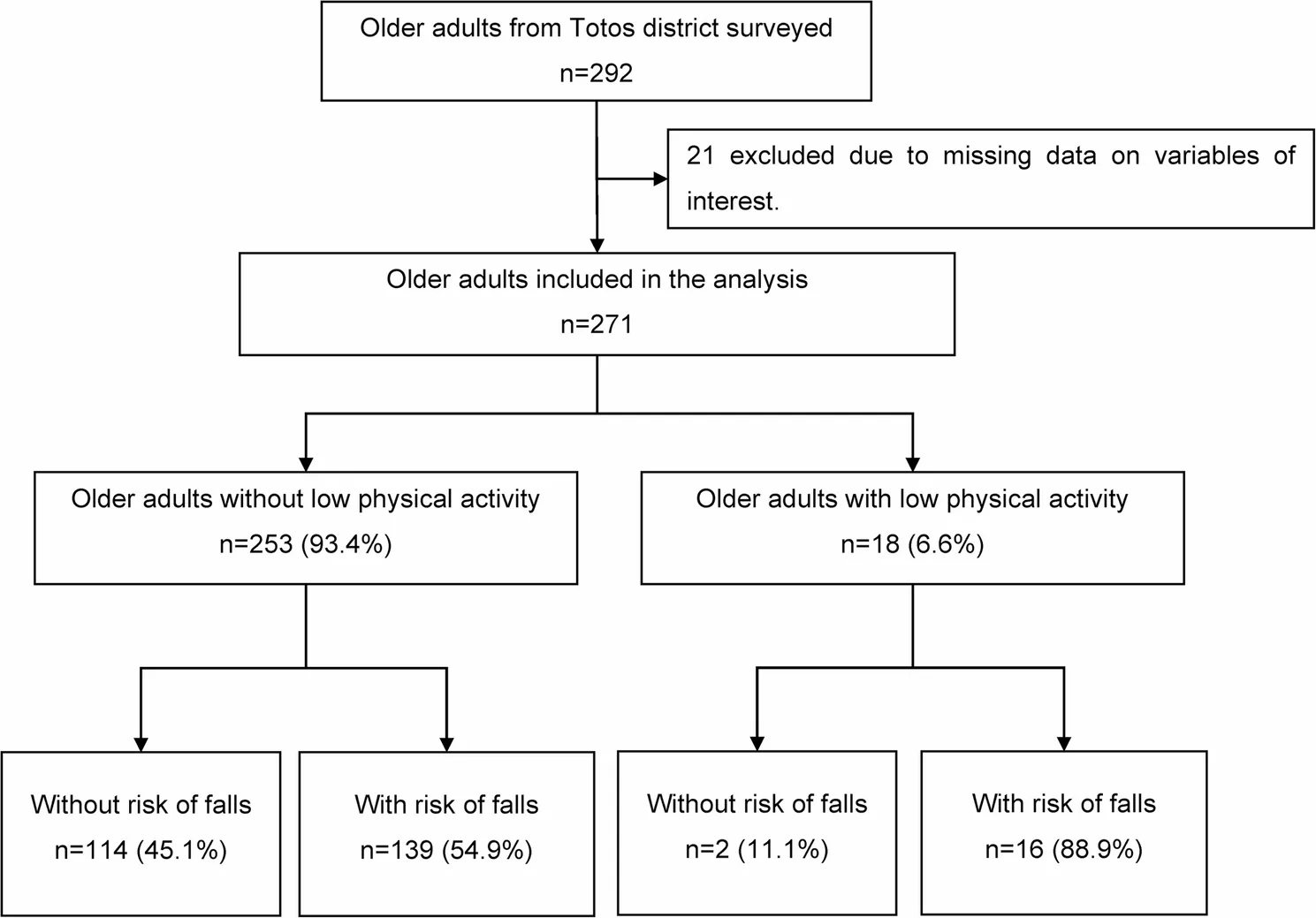
Flowchart of sample selection

After excluding 21 participants with missing data (Fig. 1), we included 271 older adults. Their median age was 75 years (interquartile range 68–81), 59.8% (*n* = 162) were women and 62.0% (*n* = 168) were cohabiting or married. Furthermore, 83.0% (*n* = 225) had no education or had not completed elementary school. Additionally, 71.6% (*n* = 194) had cognitive impairment, 57.6% (*n* = 156) presented depressive symptoms, and 30.3% (*n* = 82) had risk of sarcopenia. We found that 6.6% (*n* = 18) had low physical activity, while 57.2% (*n* = 155) had risk of falls. There was a higher prevalence of low physical activity among older adults with risk of falls (10.3% vs. 1.7%; *p* = 0.005).

Low physical activity was associated with advanced age, with a median age of 85 years compared to 74 years among those who were physically active (*p* < 0.001). When examining age categories, the prevalence of low physical activity was higher among those aged 80 years or older (17.8% vs. 2.5% among those aged 60–80 years, *p* < 0.001). Cognitive impairment was also associated with low physical activity; those with moderate impairment had a prevalence of 12.2% compared to 1.3% among those without impairment (*p* = 0.003). Similarly, risk of sarcopenia was more prevalent among those with low physical activity (20.7% vs. 0.5%, *p* < 0.001). The descriptive and bivariate analyses according to low physical activity are shown in Table 1.

**Table 1:** Descriptive and bivariate analysis of variables according to physical activity level in older adults residing in a high-Andean district of Ayacucho, Peru (*n* = 271)

Variables	Total		Low physical activity				*P* value
			No		Yes		
	*n*	%	*n* = 253	80.5	*n* = 18	6.6	
Sex							0.265
Female	162	59.8	149	92.0	13	8.0	
Male	109	40.2	104	95.4	5	4.6	
Age, median (IQR)			74	67–80	85	80–89	< 0.001
Age groups							< 0.001
60 to 80 years	198	73.1	193	97.5	5	2.5	
Older than 80 years	73	26.9	60	82.2	13	17.8	
Marital status							0.505
Single	16	5.9	15	93.8	1	6.2	
Cohabiting or married	168	62.0	159	94.6	9	5.4	
Widowed or divorced	87	32.1	79	90.8	8	9.2	
Educational level							0.418
No education/Incomplete elementary	225	83.0	207	92.0	18	8.0	
Complete primary/Incomplete secondary	28	10.3	28	100.0	0	0.0	
Complete secondary school	12	4.4	12	100.0	0	0.0	
Technical or professional education	6	2.2	6	100.0	0	0.0	
Comorbidities							0.118
0	140	51.7	131	93.6	9	6.4	
1	74	27.3	66	89.2	8	10.8	
2 or more	57	21.0	56	98.3	1	1.8	
High blood pressure	39	14.4	37	94.9	2	5.1	1.000
Type 2 diabetes mellitus	16	5.9	16	100.0	0	0.0	0.610
Chronic kidney disease	14	5.2	13	92.9	1	7.1	1.000
Arthritis (including osteoarthritis and rheumatoid arthritis)	32	11.8	30	93.8	2	6.3	1.000
Hypercholesterolemia	39	14.4	35	89.7	4	10.3	0.305
Gastritis	22	8.1	21	95.5	1	4.5	1.000
Low back pain	17	6.3	17	100.0	0	0.0	0.613
Osteoporosis	11	4.1	11	100.0	0	0.0	1.000
Polypharmacy							0.388
No	246	90.8	228	92.7	18	7.3	
Yes	25	9.2	25	100.0	0	0.0	
Body mass index, mean ± SD			25.9	3.7	25.8	4.5	0.901
Cognitive impairment							0.003
No	77	28.4	76	98.7	1	1.3	
Mild	71	26.2	69	97.2	2	2.8	
Moderate	123	45.4	108	87.8	15	12.2	
Depressive symptoms							0.193
No	115	42.4	110	95.7	5	4.3	
Yes	156	57.6	143	91.7	13	8.3	
Urinary incontinence							0.148
No	135	49.8	129	95.6	6	4.4	
Yes	136	50.2	124	91.2	12	8.8	
Risk of sarcopenia							< 0.001
No	189	69.7	188	99.5	1	0.5	
Yes	82	30.3	65	79.3	17	20.7	
Risk of falls							0.005
No	116	42.8	114	98.3	2	1.7	
Yes	155	57.2	139	89.7	16	10.3	

*IQR* Interquartile range, *SD* Standard deviation

### Descriptive and bivariate analysis according to the prevalence of risk of falls in the study sample

Descriptive and bivariate analyses showed that risk of falls was associated with several factors. Low physical activity was more prevalent among those with risk of falls (88.9% vs. 54.9%, *p* = 0.005), and females exhibited higher risk of falls compared to males (66.1% vs. 44.0%, *p* < 0.001). Older age was associated with greater risk of falls, with a median age of 78 years among those at risk compared to 71 years among those without risk (*p* < 0.001). When examined by age groups, those aged 80 years or older showed a higher prevalence of fall risk (75.3% vs. 50.5% among those aged 60–80 years, *p* < 0.001).

Regarding marital status, widowed or divorced individuals were more likely to have risk of falls (71.3% vs. 49.4% among cohabiting or married, *p* = 0.003). Educational level showed a gradient, with higher risk of falls among those with no education or incomplete elementary school (61.8%) compared to those with complete secondary or higher education (33.3%, *p* = 0.005). Cognitive impairment was associated with higher risk of falls; those with moderate impairment had a prevalence of 68.3% compared to 41.6% among those without impairment (*p* = 0.001). Risk of sarcopenia was associated with risk of falls (75.6% vs. 49.2%, *p* < 0.001), as was osteoporosis (90.9% vs. 56.2%, *p* = 0.027) (Table 2).

**Table 2:** Descriptive and bivariate analysis of variables according to risk of falls in older adults residing in a high-Andean district of Ayacucho, Peru (*n* = 271)

Variables	Risk of falls				*P* value
	No		Yes		
	*n*	%	*n*	%	
	116	42.8	155	57.2	
Low physical activity					0.005
No	114	45.1	139	54.9	
Yes	2	11.1	16	88.9	
Sex					< 0.001
Female	55	33.9	107	66.1	
Male	61	56.0	48	44.0	
Age, median (IQR)	71	66–78	78	72–83	< 0.001
Age groups					< 0.001
60 to 80 years	98	49.5	100	50.5	
Older than 80 years	18	24.7	55	75.3	
Marital status					0.003
Single	6	37.5	10	62.5	
Cohabiting or married	85	50.6	83	49.4	
Widowed or divorced	25	28.7	62	71.3	
Educational level					0.005
No education/Incomplete elementary	86	38.2	139	61.8	
Complete primary/Incomplete secondary	18	64.3	10	35.7	
Complete secondary school	7	58.3	5	41.7	
Technical or professional education	5	83.3	1	16.7	
Comorbidities					0.762
0	61	43.6	79	56.4	
1	33	44.6	41	55.4	
2 or more	22	38.6	35	61.4	
High blood pressure	14	35.9	25	64.1	0.346
Type 2 diabetes mellitus	7	43.8	9	56.3	1.000
Chronic kidney disease	5	35.7	9	64.3	0.783
Arthritis (including osteoarthritis and rheumatoid arthritis)	14	43.8	18	56.3	0.908
Hypercholesterolemia	12	30.8	27	69.2	0.101
Gastritis	12	54.6	10	45.5	0.246
Low back pain	15	88.2	2	11.8	< 0.001
Osteoporosis	1	9.1	10	90.9	0.027
Polypharmacy					0.056
No	110	44.7	136	55.3	
Yes	6	24.0	19	76.0	
Body mass index, mean ± SD	25.8	3.5	26.0	3.9	0.780
Cognitive impairment					0.001
No	45	58.4	32	41.6	
Mild	32	45.1	39	54.9	
Moderate	39	31.7	84	68.3	
Depressive symptoms					0.761
No	48	41.7	67	58.3	
Yes	68	43.6	88	56.4	
Urinary incontinence					0.587
No	60	44.4	75	55.6	
Yes	56	41.2	80	58.8	
Risk of sarcopenia					< 0.001
No	96	50.8	93	49.2	
Yes	20	24.4	62	75.6	

*IQR* Interquartile range, *SD* Standard deviation

### Crude and adjusted prevalence ratios to estimate the association between low physical activity and risk of falls in the study sample

The crude regression model showed that the prevalence of risk of falls was 62% higher among older adults with low physical activity (cPR = 1.62; 95%CI: 1.33–1.97; *p* < 0.001). After adjustment for sex, age group, marital status, educational level, comorbidities and cognitive impairment, low physical activity was associated with a 32% higher prevalence of risk of falls (aPR = 1.32; 95% CI: 1.09–1.62; *p* = 0.007; Table 3).

**Table 3:** Crude and adjusted regression models to estimate the association between low physical activity and risk of falls in older adults residing in a high-Andean district of Ayacucho, Peru

Low physical activity	Crude model			Adjusted model*		
	cPR	95% CI	*P* value	aPR	95% CI	*P* value
No	Reference	—	—	Reference	—	—
Yes	1.62	1.33–1.97	< 0.001	1.32	1.09–1.62	0.007

*cPR* Crude prevalence ratio, *aPR* Adjusted prevalence ratio, *CI* Confidence interval

*Adjusted for sex, age group, marital status, educational level, comorbidities, and cognitive impairment

## Discussion

### Main findings

This study included 271 older adults from the high-Andean district of Totos, Peru. We found that approximately one in ten showed low physical activity, while six in ten were at risk of falls. Low physical activity was associated with higher risk of falls compared to those who were more physically active. This association remained significant after adjusting for important confounders such as sex, age, marital status, educational level, comorbidities, and cognitive impairment.

### Comparison with previous studies

Our results align with studies from Brazil, Chile, and the United States showing that older adults who engage in more physical activity have lower risk of falls [26, 38, 39]. However, those studies were mostly conducted in urban, sea-level populations using different assessment tools, including scales for risk of falls and physical fitness tests. These methodological and contextual differences may influence the magnitude of associations, though the direction of effect is consistent across settings. In Peru, nearly half of adults do not achieve recommended physical activity levels [40], but patterns differ notably between coastal urban areas and rural Andean districts. In the Andes, daily life often involves walking on irregular terrain, carrying loads, and performing agricultural work [41]. The Totos context provides an important contrast with frequently studied urban settings and helps address a geographic evidence gap for high-altitude rural populations.

### Results interpretation

Aging combined with low physical activity is closely linked to reduced muscle strength and functional capacity. Low physical activity is associated with decreased muscle cross-sectional area and power output, progressive sarcopenia, and reduced bone mineral density through diminished mechanical loading, all of which compromise postural stability and the ability to recover from balance perturbations [40]. In our sample, the prevalence of sarcopenia risk of 30.3%, assessed by SARC-F, supports the biological plausibility of this pathway in the Totos population. Additionally, chronic hypobaric hypoxia at altitude may potentiate muscle mass loss through tissue hypoxia and autonomic cardiovascular alterations [14, 15], creating an additional vulnerability that may not be present in sea-level populations. Research from the United States suggests that routine household activities alone may be insufficient to counteract these effects over time [37]. Many older adults in Totos engage in physically demanding agricultural work [41], which may provide some protection while simultaneously exposing them to environmental hazards like steep slopes and uneven surfaces.

Women and older age groups in our sample showed higher prevalence of being at risk of falls, a pattern reported internationally [38, 39, 42]. Although we did not study sex and age as effect modifiers, their well-established associations with risk of falls support their inclusion as adjustment variables in our models. Aging affects multiple systems relevant to risk of falls: reduced capillarization in skeletal muscle decreases endurance and voluntary control [43, 44]; vision changes impair depth perception and detection of ground irregularities [45, 46]; and proprioceptive alterations challenge balance [47, 48]. Regular physical activity helps maintain these systems, reinforcing its role in comprehensive fall prevention strategies [36].

The association between physical activity and fall risk is complex and not necessarily linear. Klenk et al. [49] found no significant association between physical activity and falls per person-year in 1,214 community-dwelling older Germans; however, when falls were expressed per 100 h of gait, low-active individuals showed consistently higher rates, with the highest rates in those with slow gait speed. This highlights that the metric used to express fall risk influences the observed association. Orwoll et al. [50], in 2,741 older men, found that while the highest predicted fall rates occurred in men with low activity and low physical performance, consistent with our findings, most falls in absolute terms occurred in groups with relatively higher activity levels, showing that falls are not exclusive to the least active older adults. Peeters et al. [51] tested for a U-shaped association in 1,337 community-dwelling older adults and found no statistically significant evidence of non-linearity, with higher activity linearly associated with reduced recurrent fall risk. In the present study, physical activity was operationalized as a binary exposure based on sex-specific PASE cutoffs, which limits formal testing of non-linearity. Evaluating PASE as a continuous variable was not statistically feasible given only 18 participants in the low-activity group. Whether a U-shaped or more complex pattern exists in high-Andean populations, where higher activity often involves exposure to rugged terrain, requires investigation in future studies with larger samples.

FoF is a psychological factor not directly measured in this study, which can independently reduce physical activity, restrict mobility and social participation, and contribute to depressive symptoms, thereby creating a cycle that further increases fall risk [52]. Its global prevalence among older adults reaches nearly 50%, with higher rates reported in developing countries [53]. Although FoF was not assessed in our study, 57.6% of participants presented depressive symptoms, a condition closely associated with FoF in older adults. Future studies in high-Andean communities should incorporate validated instruments for FoF assessment, such as the Falls Efficacy Scale-International (FES-I), to evaluate its role in the association between physical activity and fall risk.

A notable finding in our data is that among the 18 participants with low physical activity, only one had no cognitive impairment, while 15 presented moderate cognitive impairment. This pattern is consistent with evidence linking cognitive impairment, particularly executive dysfunction, to reduced motivation and impaired planning of daily activities, which may translate into lower physical engagement [54]. This overlap also suggests that cognitive decline and low physical activity may mutually reinforce each other, further contributing to fall risk [55].

### Environmental and infrastructural perspective

The environmental context of the Peruvian Andes is central to understanding risk of falls. Roads and footpaths are often stony, narrow, and steep, with limited lighting, especially at night or during the rainy season [56]. These conditions increase exposure to slips and trips, even for individuals with good strength and balance. Many high-Andean communities are located far from healthcare facilities, and travel to clinics may require long walks or multiple transport segments [56]. Remote rural areas face constraints in infrastructure and timely access to health services, which can delay care after falls or near-falls and may discourage older adults from seeking preventive advice.

Interventions should address both individual capacity and environmental risk. Community-led efforts could improve lighting, install handrails on steep paths, and reduce hazards on common walking routes. Home hazard screening—identifying loose rugs, cluttered passageways, or unsafe stairs—should be combined with simple, affordable modifications using local materials [56].

### Strengths and limitations

This study has important strengths. We conducted a district-wide census of older adults in Totos, achieving high coverage of the target population through coordination with municipal authorities and community representatives. Our data collection team received standardized training, and assessments were conducted by staff fluent in Spanish and Quechua Chanka, ensuring culturally appropriate data collection. We used validated instruments for all key measurements, including the PASE and the Functional Reach Test. Our multivariable models adjusted for prespecified covariates identified from prior literature, improving comparability with other studies [26, 38, 39, 42].

Several limitations should be noted. The cross-sectional design prevents establishing temporal ordering between activity level and risk of falls. Census-based non-probabilistic sampling limits generalizability beyond similar rural Andean settings. Some measures were self-reported, which may introduce recall error. The Functional Reach Test was obtained with a single measurement, whereas some protocols recommend multiple attempts for reliability. Additionally, the Functional Reach Test cutoff of 20.32 cm was originally derived from sea-level populations and its applicability to high-Andean communities warrants further validation, and the use of different cutoffs across studies may limit direct comparability of results [57]. The PASE captures self-reported physical activity but does not quantify sedentary time or provide objective measurements of activity intensity. Celis-Morales et al. [13] showed that self-reported measures can underestimate the strength of associations with health outcomes by 43–50% compared to accelerometry. Future studies should consider incorporating accelerometers in this population. The small number of participants classified as having low physical activity (*n* = 18) may affect the precision of stratum-specific estimates. While the Poisson regression approach models the outcome as a function of the exposure and covariates directly, results should be interpreted with caution and replication in larger samples from similar high-Andean settings is needed to confirm these findings. The high concurrent prevalence of cognitive impairment, depressive symptoms, and sarcopenia risk should be acknowledged, as these conditions are independently associated with both lower physical activity and higher fall risk and may have influenced our estimates. Cognitive impairment was included in the adjusted model; depressive symptoms and sarcopenia risk were not, given their potential role as mediators in the pathway between physical activity and fall risk rather than as confounders. Finally, while we adjusted for multiple covariates, unmeasured factors such as vision acuity, specific medications, home environment details, or history of previous falls may contribute to residual confounding [36, 43–48, 56, 58].

## Conclusions

In this high-Andean community, low physical activity was associated with higher risk of falls among older adults. These findings can inform early intervention strategies in primary care and community settings. Feasible screening tools like PASE and the Functional Reach Test can help identify older adults at risk of falls in resource-constrained clinics and support the development of locally adapted physical activity programs in rural high-altitude areas. 

## Data Availability

The datasets utilized and analyzed in the present study are accessible upon reasonable request to the corresponding author.

## Abbreviations

IPAQ: International Physical Activity Questionnaire
PASE: Physical Activity Scale for the Elderly
M.a.s.l.: Meters above sea level
STROBE: Strengthening the Reporting of Observational Studies in Epidemiology
BMI: Body Mass Index
HBP: High blood pressure
T2DM: Type 2 Diabetes Mellitus
CKD: Chronic Kidney Disease
SPMSQ: Short Portable Mental Status Questionnaire
GDS-5: 5-item Geriatric Depression Scale
SARC-F: Strength, Assistance in walking, Rise from a chair, Climb stairs, and Falls
REDCap: Research Electronic Data Capture
95% CI: 95% Confidence Interval
cPR: Crude Prevalence Ratio
aPR: Adjusted Prevalence Ratio
VIF: Variance Inflation Factor
